# *Hippocampus nalu*, a new species of pygmy seahorse from South Africa, and the first record of a pygmy seahorse from the Indian Ocean (Teleostei, Syngnathidae)

**DOI:** 10.3897/zookeys.934.50924

**Published:** 2020-05-19

**Authors:** Graham Short, Louw Claassens, Richard Smith, Maarten De Brauwer, Healy Hamilton, Michael Stat, David Harasti

**Affiliations:** 1 Research Associate, Ichthyology, Australian Museum Research Institute, Sydney, Australia Australian Museum Research Institute Sydney Australia; 2 Research Associate, Ichthyology, California Academy of Sciences, San Francisco, USA Ichthyology, California Academy of Sciences San Francisco United States of America; 3 Research Associate, Ichthyology, Burke Museum, Seattle, USA Burke Museum Seattle United States of America; 4 IUCN Seahorse, Pipefish Stickleback Specialist Group, University of British Columbia, Vancouver, Canada University of British Columbia Vancouver Canada; 5 Rhodes University, Grahamstown, South Africa Rhodes University Grahamstown South Africa; 6 Knysna Basin Project, Knysna, South Africa Knysna Basin Project Knysna South Africa; 7 University of Leeds, Leeds, UK University of Leeds Leeds United Kingdom; 8 NatureServe, Arlington, Virginia, USA NatureServe Arlington United States of America; 9 University of Newcastle, Callaghan, NSW, Australia University of Newcastle Callaghan Australia; 10 Port Stephens Fisheries Institute, NSW, Australia Port Stephens Fisheries Institute Anna Bay Australia

**Keywords:** Africa, COI, cryptobenthic, ichthyology, marine fish, morphology, Sodwana Bay, taxonomy

## Abstract

A new species and the first confirmed record of a true pygmy seahorse from Africa, *Hippocampus nalu***sp. nov.**, is herein described on the basis of two specimens, 18.9–22 mm SL, collected from flat sandy coral reef at 14–17 meters depth from Sodwana Bay, South Africa. The new taxon shares morphological synapomorphies with the previously described central Indo-Pacific pygmy seahorses, *H. colemani*, *H. japapigu*, *H. pontohi*, and *H. satomiae*, and *H. waleananus*, including diminutive size, twelve trunk rings, prominent cleithral ring and supracleithrum, spines on the fifth and twelfth superior and lateral trunk ridges, respectively, and prominent wing-like protrusions present on the first and/or second superior trunk rings posterior to the head. *Hippocampus nalu***sp. nov.** is primarily distinguished from its pygmy seahorse congeners by highly distinct spine morphology along the anterior segments of the superior trunk ridge. Comparative molecular analysis reveals that the new species demonstrates significant genetic divergence in the mitochondrial COI gene from the morphologically similar *H. japapigu* and *H. pontohi* (estimated uncorrected p-distances of 16.3% and 15.2%, respectively). *Hippocampus nalu***sp. nov.** represents the eighth member of the pygmy seahorse clade to be described from the Indo-Pacific, the first confirmed record from the African continent and the Indian Ocean, and an extension of more than 8000 km beyond the previously known range of pygmy seahorses from the Central and Western Indo-Pacific.

## Introduction

The family Syngnathidae contains more than 300 species within 57 genera of predominantly small-bodied and cryptic marine fishes (Dawson 1985; Hamilton et al. 2017). The family is widely distributed in temperate and tropical habitats among mostly shallow coastal areas of the Atlantic and Indo-Pacific Oceans, including soft sediment habitats, seagrass beds, estuaries, coral and rocky reefs, and mangroves (Foster and Vincent 2004; Kuiter 2009). Members of this family, comprising the seahorses, pipefishes, pipehorses, and seadragons, are uniquely characterised by a fused jaw that allows for alimentation by suction feeding, male brooding, and cryptic morphology and behaviour. Seahorses cluster into pygmy and non-pygmy phylogenetic lineages (Hamilton et al. 2017). Pygmy seahorses are generally diminutive in size, less than 27.3 mm SL, live in obligate association with octocorals or occur freely in clumps of calcareous algae, hydroids or algal turf, and are distinct from the more speciose group of larger (24–350mm SL) non-pygmy species of *Hippocampus* in possessing a single rather than paired gill openings and trunk brooding of their young (Whitley 1970; Kuiter 2003; Lourie and Randall 2003; Lourie and Kuiter 2008; Gomon and Kuiter 2009; Lourie et al. 2016; Short et al. 2018). Seven pygmy seahorse species are currently recognized: *H. bargibanti* Whitley, 1970, *H. denise* Lourie & Randall, 2003; *H. colemani* Kuiter, 2003; *H. pontohi* Lourie & Kuiter, 2008; *H. satomiae* Gomon & Kuiter, 2009; *H. waleananus* Gomon & Kuiter, 2009; and *H. japapigu*Short et al., 2018. The generally free-living *H. colemani*, *H. japapigu*, *H. pontohi*, and the soft and octocoral associating *H. satomiae* and *H. waleananus*, respectively, are united by similar meristic and morphometric characters, conserved morphology, and a suite of synapomorphic characters, including twelve trunk rings, prominent cleithra and supracleithrum, diagnostic spines on the fifth and twelfth superior and lateral trunk ridges, respectively, and prominent wing-like protrusions present on the first and/or second superior trunk rings posterior to the head (Short et al. 2018). In contrast, the gorgonian octocoral-dwelling species, *H. bargibanti* and *H. denise*, are distinct in overall morphological appearance in having an indiscernible coronet, indistinct cleithra, and the presence of large bulbous tubercles.

Pygmy seahorses have previously been recorded throughout the central Indo-Pacific with largely sympatric distributions, ranging from Thailand, Indonesia, Philippines, Palau, Papua New Guinea, Australia, Solomon Islands, New Caledonia, Vanuatu, Fiji, to Taiwan and central Japan (Whitley 1970; Kuiter 2003; Lourie and Randall 2003; Senou et al. 2006, 2007, 2008; Baine and Harasti 2007; Lourie and Kuiter 2008; Motomura et al. 2010; Allen and Erdmann 2012; Smith et al. 2012; Lourie et al. 2016; Short et al. 2018; Heard et al. 2019) with no species heretofore recorded in the Western Indo-Pacific. In 2017, Dive Instructor Savannah Nalu Olivier (Pisces Diving, Sodwana Bay, South Africa), during the course of a scuba dive with beginner divers, observed and photographed multiple individuals of a diminutive seahorse from flat sandy-algal reef habitat in gullies with strong surge currents in Sodwana Bay, South Africa. Subsequent investigation revealed that these diminutive seahorses, although similar to *H. pontohi* and *H. japapigu* in appearance and colouration, differed markedly in a number of characters, and represent a new species and the first confirmed record of a true pygmy seahorse from South Africa, the African continent, and the Indian Ocean, which is described herein.

## Materials and methods

Two specimens of *H. nalu* were collected by hand while scuba diving in 12–17 m depth. The holotype (SAMC-F04193) and paratype (SAMC-F04194) were deposited in the fish collection of the Iziko South African Museum (SAM), Cape Town, South Africa. Head and body measurements follow Short et al. (2018) and are expressed as percent of standard length (SL) or head length (HL). Computed tomography (CT) scans of both specimens were obtained at the University of New England, NSW, Australia CT Scanner Facility using a GE v|tome|x S industrial micro-CT scanner with a voxel resolution (slice thickness) of 8 µm. The resulting CT scan data were visualized and rendered in Horos (http://www.horosproject.org). All digital images were processed using Adobe Photoshop.

Genomic DNA was extracted from the right eye of the 95% ethanol-fixed holotype of *H. nalu*SAM-MB-F041933) using a DNeasy Blood and Tissue Extraction Kit (Qiagen, Inc.) in accordance with the manufacturer’s protocols and initial tissue digestion overnight at 56 °C. A shotgun library was prepared from the extracted DNA using an Illumina Nextera DNA Flex library prep kit and sequenced using a MiSeq and 2×150 v3 reagent kit at the Ramaciotti Centre for Genomics (UNSW Sydney, Australia).

The mitochondrial genome was assembled by initially mapping reads to a reference 655 bp COI fragment from *Hippocampus kuda* (Genbank: EU930325) in Geneious v 10.05. When no reads could be further extracted from the shotgun dataset, sequences with a minimum average phred quality score of 30 were filtered out using BBDuk and used to assemble the complete mitochondrial genome with the De Novo Assemble function in Geneious. The mitogenome was annotated by comparison to the *Hippocampus kuda* mitochondrial genome (AP005985) and the Find ORFs function in Geneious using the Vertebrate Mitochondrial translation table. The complete mitochondrial genome of *H. nalu* is available from GenBank under accession number MT053858.

Genetic distances (uncorrected *p*-distances) were calculated based on COI using MEGA v. 7.0.26 (Kumar et al. 2017).

## Systematics

### Hippocampus nalu sp. nov.

Taxon classificationAnimaliaSyngnathiformesSyngnathidae

A1E8EF2B-B627-5C88-9B5D-C8AE088DA071

http://zoobank.org/FE8B30CB-3672-45DD-AF96-B4FA574979BE

**Figure 1.**
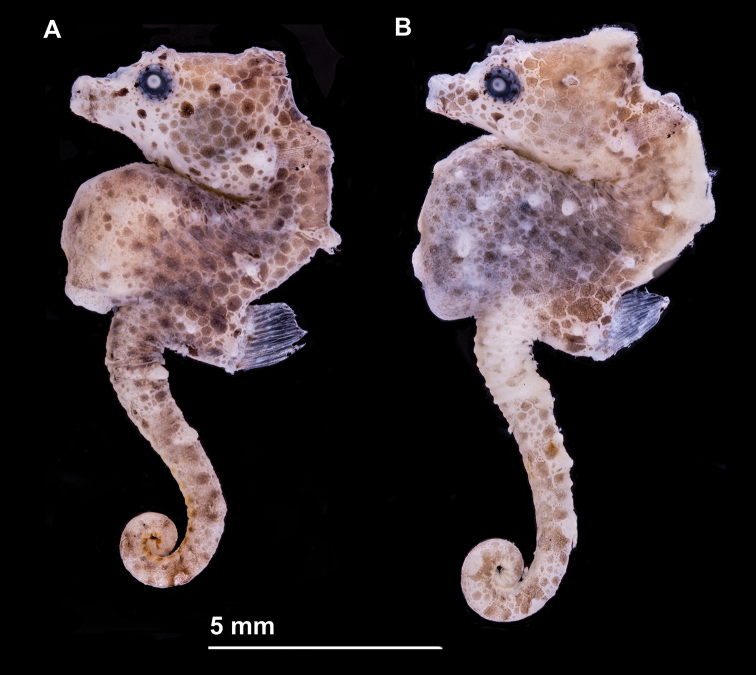
*Hippocampus nalu*, preserved specimens **A** SAMC-F041933, holotype female, 18.9 mm SL, and **B** SAMC-F041934, paratype, male, 22 mm SL; South Africa: Sodwana Bay, 2 Mile Reef (photograph Australian Museum Research Institute).

**Figure 2.**
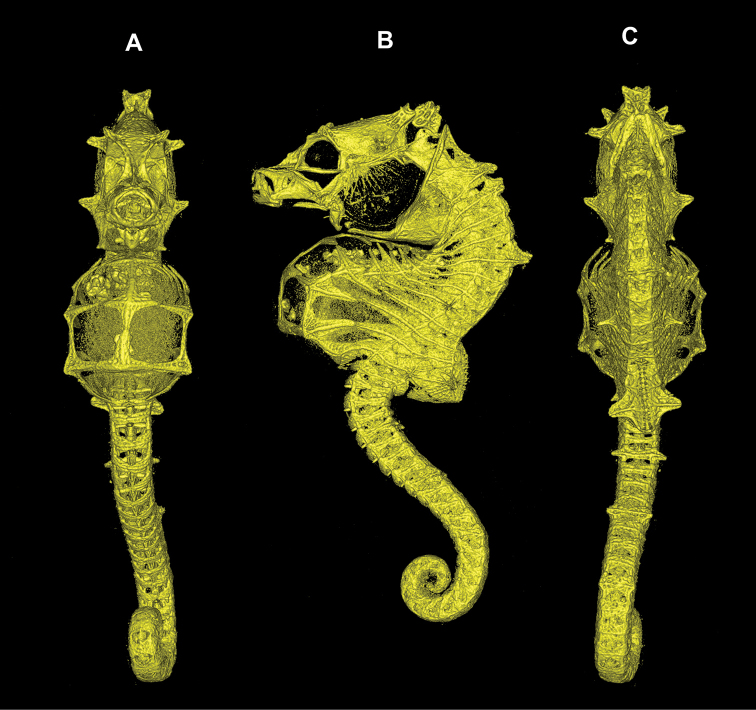
Computed tomography scanned skeleton of *Hippocampus nalu*, SAMC-F041933, holotype, 18.9 mm SL, female SL**A** ventral view **B** lateral view **C** dorsal view.

**Figure 3.**
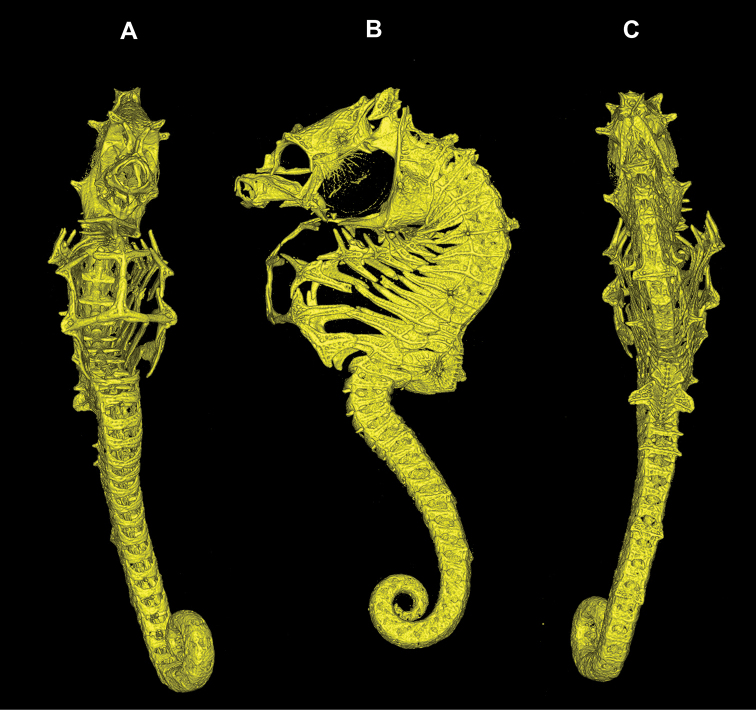
Computed tomography scanned skeleton of *Hippocampus nalu*, SAMC-F041934, paratype, male, 22 mm SL**A** ventral view **B** lateral view **C** dorsal view.

**Figure 4.**
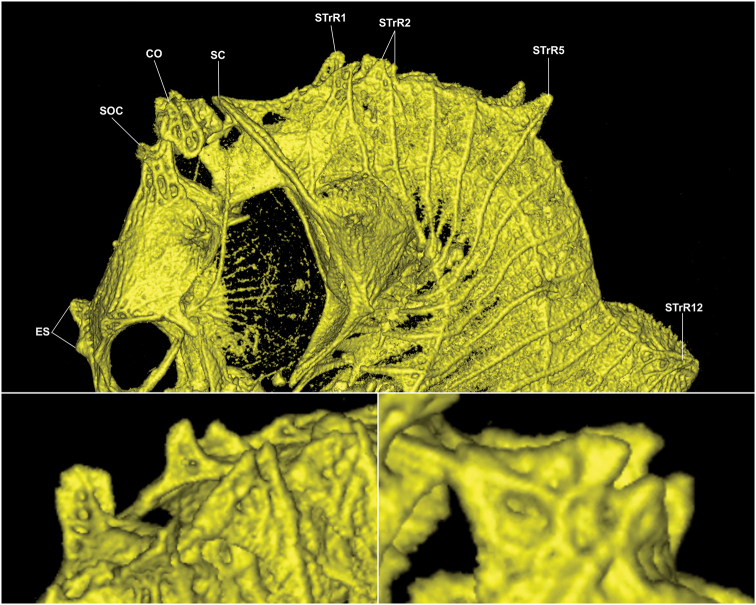
Computed tomography scan of *Hippocampus nalu*, SAMC-F041933, holotype, female, 18.9 mm SL, 2 Mile Reef, Sodwana Bay, South Africa **A** lateral view of head and trunk area **B** anterolateral view of first and second superior trunk ridge spines (STrR1-2) **C** close-up lateral view of second superior trunk ridge cuspidate spines (STrR2). Abbreviations: ES, double eye spines; SOC, supraoccipital; CO, coronet; SC, supracleithrum; STrR1, first superior trunk ridge spine; STrR2, second superior trunk ridge spines; STrR5, fifth superior trunk ridge spine; STrR12, twelfth superior trunk ridge spine.

**Figure 5.**
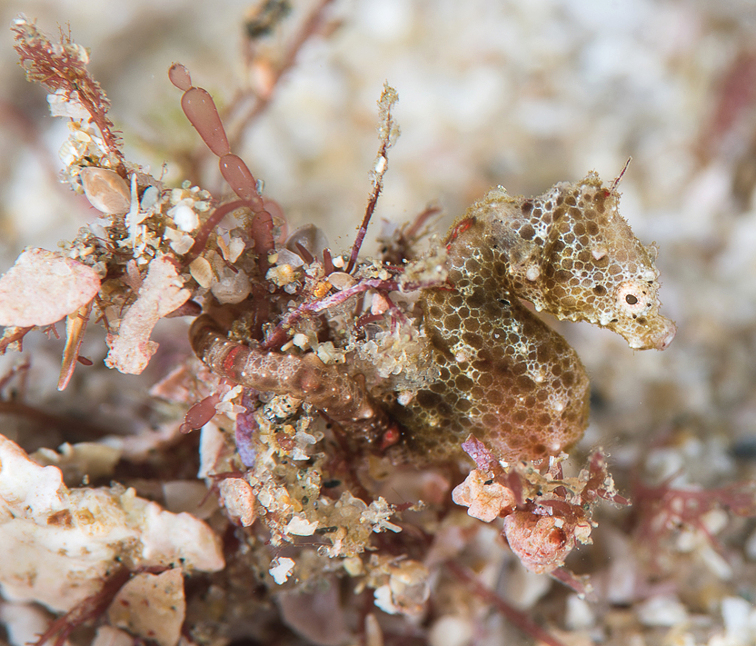
*Hippocampus nalu* in situ, SAMC-F041933, holotype, female, Sodwana Bay, South Africa at 14 m depth (photograph Richard Smith / oceanrealmimages.com).

**Figure 6.**
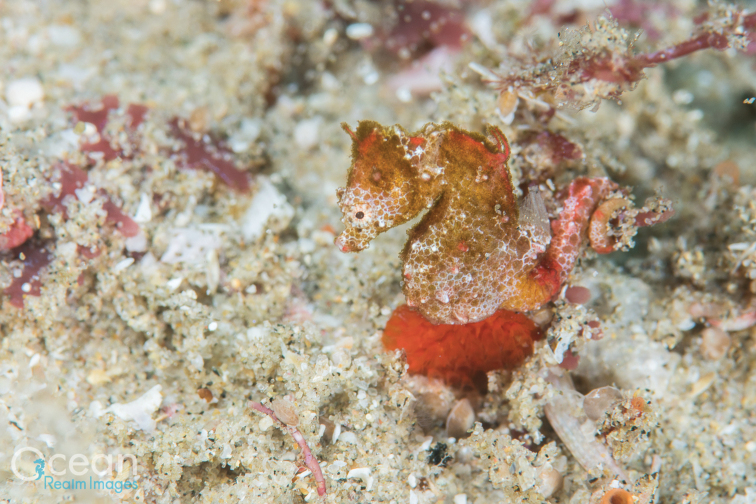
*Hippocampus nalu* in situ, SAMC-F041934, paratype, male, Sodwana Bay, South Africa at 14 m depth (photograph Richard Smith / oceanrealmimages.com).

Figures 1 , 2 , 3 , 4 , 5 , 6 , Tables 1 , 2

#### Type locality.

2 Mile Reef, Sodwana Bay, South Africa, 27°30'46.6"S, 32°41'10.4"E.

#### Holotype.

**SAMC-F041933**, 18.9 mm SL, female, 2 Mile Reef, Sodwana Bay, South Africa, 27°30'46.6"S, 32°41'10.4"E, 12–17 meters depth, 18 October 2018, L. Claassens, R. Smith, S. Olivier, scuba diving.

#### Paratype.

**SAMC-F041934**, 22 mm SL, male, 2 Mile Reef, Sodwana Bay, South Africa, 27°30'46.6"S, 32°41'10.4"E, 12–17 meters depth, 18 October 2018, L. Claassens, R. Smith, S. Olivier, scuba diving.

#### Comparative material.

Published data was obtained for *H. japapigu* (Short et al. 2018); *H. pontohi* and *H. satomiae* (Lourie and Kuiter 2008); *H. colemani* (Kuiter (2003); and *H. waleananus* (Gomon and Kuiter 2009).

#### Diagnosis.

*Hippocampus nalu* sp. nov. is diagnosed by the following combination of characters: tail rings 29–30; dorsal fin rays twelve; pectoral fin rays ten; subdorsal rings four; two pairs of bilateral wing-like protrusions behind the head formed by a pair of large oblong spines projecting anterolaterad on the first superior trunk ridge and a pair of unique double cuspidate spines projecting anteriad on the second superior trunk ridge; double spine above the eyes; absence of spines at the sixth superior trunk and eighth inferior trunk ridges; superior trunk ridge ending with two subdorsal spines protruding laterad; the posteriormost spine greatly enlarged on twelfth trunk ridge.

**Table 1.** Comparison of relative measurements and counts between *H. nalu*, *H. japapigu*, *H. pontohi*, *H. colemani*, *H. satomiae*, and *H. waleananus* based on type specimens.

	***H. nalu***	***H. nalu***	***H. japapigu***	***H. pontohi***	***H. colemani***	***H. satomiae***	***H. waleananus***
Voucher	SAMC F041933	SAMC F041934	Short et al. 2018	Lourie and Kuiter 2008	Kuiter 2003	Lourie and Kuiter 2008	Gomon and Kuiter 2009
SL (mm)	18.9	22.0	16.3	16.7	26.9	13.6	17.8
% SL							
Snout depth	86.7	85.1	74.0	84.2	70.5	86.0	95.2
HL	23.5	20.3	18.0	21.7	18.1	22.0	17.7
Trunk length	29.9	30.9	32.6	33.3	32	30.0	31.3
Tail length	46.5	48.9	49.4	45.0	50.0	48.0	63.4
Trunk depth at dorsal-fin origin	27.1	28.1	18.7	13.5	19.2	13.0	15.0
% HL							
Coronet height	48.6	51.2	58.1	47.4	45.6	40.2	48.3
Head depth	63.6	69.1	69.9	60.6	62.6	51.8	67.9
Snout length	25.4	25.6	28.7	23.2	27.7	27.0	26.8
Post-orbital length	56.2	54.7	55.3	51.2	52.1	45.0	51.5
Trunk rings	12	12	12	12	12	12	12
Tail rings	29	30	28	28–30	26–28	27–28	32
Dorsal fin rays	12	12	14	12	14	13	12
Pectoral fin rays	10	10	9	9–10	9	9	9

#### Description.

General body shape as in Figs 1–6. Morphometric, meristic, and morphological characters listed in Tables 1, 2. Distinct, angular coronet; single gill-opening on midline behind coronet supported by prominent cleithral ring and supracleithrum; dorsal fin rays twelve; pectoral-fin rays ten; anal fin rays four; trunk rings twelve; dorsal fin base strongly raised dorsally; subdorsal rings four, dorsal fin base starting immediately posterior to ninth trunk ring and ending immediately posterior to first tail ring; tail rings 29–30. Body ornamentation: prominent double spine present dorsally of eye, posteriormost spine large, anteriormost spine small; lateral head spine ventral of coronet on postemporal; two moderately large spines on cleithral ring, upper spine at level of last pectoral fin ray, lower spine at ventral extent of ring; snout spine broad, on midline between eyes; superior trunk ridge with two pairs of bilateral wing-like protrusions formed by a pair of large oblong spines projecting anterolaterad on the first superior trunk ridge and a pair of unique double cuspidate spines projecting anteriad on the second superior trunk ridge; superior trunk ridge ending with two subdorsal spines protruding laterad, the posteriormost spine greatly enlarged on the twelfth trunk ridge; laterodorsal surface of trunk flat; large spines on fifth superior trunk ridge; moderate spines on fifth lateral trunk ridge, large spines on eighth lateral trunk ridge; all inferior trunk ridge spines absent; superior tail ridge spines moderately developed anteriorly, becoming smaller posteriorly, with enlarged spines on fifth, ninth, twelfth, and 16^th^ tail ridges; inferior tail ridge spines absent; caudal fin absent.

#### Colouration.

In life, *H. nalu* (Figs 5, 6) exhibits cryptic colouration: head, trunk, and tail, honey brown, with overlay of white irregular quadrilateral and pentagonal reticulation; dorsal trunk area from the third to the tenth superior trunk rings with red colouration, white reticulation absent; white reticulation on trunk ventrolaterally, dorsal fin base dorsally, neck dorsolaterally, areas of head (snout, orbitals, and operculum, and along the length of the tail; dermal appendages, thin and red, projecting anteriorly on coronet and fifth superior trunk ridges. In alcohol, head and body background colour typically uniformly pale cream to light brown (Figure 1). The type specimens have retained the reticulated colour pattern of live individuals. Fins hyaline.

#### Distribution and habitat.

*Hippocampus nalu* is currently known only from shallow (12–17 meters depth) waters in Sodwana Bay, South Africa, which falls within the iSimangaliso Wetland Park, a World Heritage Site that stretches from Lake St Lucia in the south to the Mozambique border in the north, and along the north coast of KwaZulu Natal province. However, the new species likely has a wider distribution along the East African coast and remains undetected because of its cryptic behaviour and diminutive size, and therefore its occurrence further north off East Africa in Mozambique, Tanzania, and Kenya, and offshore to Madagascar, may be confirmed by future localised ichthyofaunal surveys.

Sodwana Bay is situated near the southern end of the tropical western Indo-Pacific marine ecoregion, and is a subtropical transition zone between the tropics to the north and the warm temperate region to the south; consequently, it maintains the southernmost reef formations dominated by coral in Africa (Glassom et al. 2006). The composition and distribution of marine inshore ichthyofauna in Sodwana Bay are influenced by a complex interplay of south-flowing currents, including the Agulhas, Mozambique, and East Madagascar currents that transport warm waters to the East coast of South Africa, and are therefore likely to act as a main conduit to transport tropical fish species from East Africa and elsewhere in the Western Indo-Pacific south to Sodwana Bay (Beckley 1995, 2000). Across South Africa, there are four other species of non-pygmy seahorses known to occur: the temperate endemic *H. capensis* and the subtropical–tropical Indo-Pacific *H. camelopardalis*, *H. histrix*, and *H. kuda* (Lockyear 2006; Lourie et al. 2016; Claassens and Hodgson 2018).

The underwater ecosystems where *H. nalu* was observed consisted of flat sandstone-based coral reefs in addition to unique topographic features (Ramsay et al. 1994) comprising low pinnacles, shallow drop offs, and sandy gullies, the latter being exposed to strong currents. *Hippocampus nalu* was found loosely associating with short algal turf, used as a holdfast, which was growing on sand-covered coral bedrock separated by sandy gullies (around 2 meters wide). The ambient seawater temperature averaged approximately 24 °C during the dives, which were conducted in October of 2018. The second and third authors experienced strong swells on the exposed reefs of 2 Mile Reef during data collection. The holotype and paratype appeared to be a mated pair, which were found within approximately 60 cm distance of each other on the two dives and their behaviour was observed prior to collection. Direct interaction, however, was not observed. Behaviour was very similar to congeners *H. pontohi* and *H. japapigu* (Short et al. 2018). Multiple individuals of *H. nalu*, including a small juvenile, were found in the gullies and observed to be associated with low-growth algal turf. The small juvenile, estimated to be approximately 10 mm SL (Figure 6), retained the dark colouration of a recently settled juvenile pygmy seahorse (Smith 2010).

#### Etymology.

Named after Savannah Nalu Olivier who discovered the new species in Sodwana Bay. In the South African languages, Xhosa and Zulu, *nalu* refers to the expression ‘here it is’ and therefore we extend its meaning in this case to the simple fact that *H. nalu* was there all along until its discovery. Additionally, the species name *nalu* is also the Hawaiian word that refers to the waves or surf of the moana (ocean), for that reason we find the name relevant as *H. nalu* was observed moving about in strong surge to different locations in the sandy habitat. A noun in the genitive.

New English Names: Sodwana pygmy seahorse, African pygmy seahorse, and Honeypot seahorse are proposed here for *Hippocampus nalu*.

#### Mitochondrial genome and COI genetic distances.

A total of 7,259,439 forward and reverse reads were recovered from the shotgun library of which 13,133 were used to assemble the mitogenome of *H. nalu* (coverage: mean=117.1, SD=20.3). The mitogenome of *H. nalu* is 16,470 bp in size consisting of 13 protein-coding genes, two rRNA genes, 22 tRNA genes, and a control region (D-loop). Suppl. material 1 shows the genetic distance analysis at the COI gene (uncorrected p distances) between *H. nalu* and the previously sequenced specimens of *H. bargibanti*, *H. denise*, *H. japapigu*, and *H. pontohi* (Hamilton et al. 2017; Short et al. 2018). *Hippocampus nalu* differs from *H. pontohi* by 15.2%, from *H. japapigu* by 16.3%, *H. bargibanti* by 16.5%, and *H. denise* by 17.1%. Reported mtDNA clock rates of approximately 1.2% per million years in marine teleosts (Reece et al. 2010) indicate divergence between *H. nalu* and its congeners *H. pontohi* and *H. japapigu* approximately 12.7 and 13.6 million years ago, respectively.

**Table 2.** Comparison of morphological characters between *H. nalu*, *H. japapigu*, and *H. pontohi*.

	*H. nalu*	*H. pontohi*	*H. japapigu*
Data source	SAMC F041933-F041934	Lourie and Kuiter 2008	Short et al. 2018
Single gill opening	present		
Elevated cleithral girdle	present		
Coronet	distinct and elevated		
Cleithral spines	pectoral fin base, ventral of 1^st^ trunk ring		
Subdorsal rings	3 trunk +1 tail		
Lateral head spine	present		
Broad snout spine	present		
Eye spine dorsad	double	single	
Eye spine ventrad	single		
1^st^ superior trunk ridge spines	present		
2^nd^ superior trunk ridge spines	present	present	absent
Elevated ridge on trunk	absent		present
5^th^ superior trunk ridge spines	present		
5^th^ lateral trunk ridge spines	present		
6^th^ superior trunk ridge spines	absent	present	
8^th^ lateral trunk ridge spines	present		
8^th^ inferior trunk ridge spines	absent	present	
Subdorsal trunk ridge spines	11^th^,12^th^	10^th^,11^th^,12^th^	
Superior tail ridge spines	5^th^, 9^th^, 12^th^		
Inferior tail ridge spines	absent		

#### Comparative remarks.

*Hippocampus nalu* shares morphological synapomorphies with its congeners *H. pontohi*, *H. japapigu*, *H. colemani*, *H. satomiae*, and *H. waleananus*, including meristics, distinct coronet, 12 trunk rings, prominent cleithral ring and supracleithrum, large spines on the fifth and twelfth superior and lateral trunk ridges, respectively, large spine on the eighth lateral trunk ridge, and prominent wing-like protrusions present on the first and/or second superior trunk rings posterior to the head (Table 2, Short et al. 2018: table 10). *Hippocampus nalu* (Figs 1, 5, 6) appears to be most similar in external appearance to *H. japapigu* and *H. pontohi* (Short et al. 2018: figs 1–7). However, as revealed by CT scans, it is more similar morphologically to *H. pontohi*, primarily on the basis of wing-like protrusions formed by two pairs of large spines on the first and second superior trunk ridges (Figure 4; Short et al. 2018: fig. 10), versus a single pair of large spines restricted to the first superior trunk ridge in *H. japapigu* (Short et al. 2018: fig. 9).

*Hippocampus nalu* shares with *H. pontohi* a pair of large moderately oblong spines that project anterolaterally from the first superior trunk ridge (Figs 2–4; Short et al. 2018: fig. 10), whereas *H. nalu* differs in having a large pair of unique double cuspidate spines projecting anteriad on the second superior trunk ridge (versus large, moderately oblong spines projecting anterolaterally on the second superior trunk ridge in *H. pontohi* [Figs 2–4; Short et al. 2018: fig. 10]). The new species can be further distinguished from *H. pontohi* by the following combination of characters (Table 2): double spine above the eyes (versus single); small spine ventroposterior to eye (versus absent), absence of spines at the sixth superior trunk and eighth inferior trunk ridges (versus presence of small spines); two subdorsal spines with the posteriormost greatly enlarged on the twelfth trunk ridge (versus three subdorsal spines). *Hippocampus nalu* furthermore shares with *H. pontohi* and *H. japapigu*, as revealed by CT scans, the ring and ridge structure of larger seahorses with well-developed ossification of the skeleton, including the strong ossification of the inferior and ventral trunk area (Figs 2, 3; Short et al. 2018: fig. 8) versus the incomplete ossification of the inferior and ventral trunk ridges anteriorly in *H. bargibanti* and *H. denise* (Gomon, 1997; Lourie and Randall 2003; Gomon and Kuiter 2009).

*Hippocampus nalu* can be distinguished from *H. japapigu* (Table 2) by the absence of a distinct elevated dorsal ridge internally formed by triangular bony mounds in the anterodorsal area of the trunk directly posterior to the head (Short et al. 2018: fig. 9), double spine above the eyes (versus single); absence of spines at the sixth superior trunk and eighth inferior trunk ridges (versus presence of small spines); small spine at eighth lateral trunk ridge (versus presence of prominent spine in *H. japapigu* and a small spine in *H. pontohi*). *Hippocampus nalu* exhibits a similar colour pattern to *H. japapigu* with white reticulation over the head, neck, and trunk areas, and prominent solid red colouration over the dorsal area of the entire trunk.

*Hippocampus nalu* is similar to *H. colemani* and *H. satomiae* primarily on the basis of two pairs of large spines on the first and second superior trunk ridges, respectively (Short et al. 2018: table 10). Determination of the configuration and structure of the two pairs of large spines for *H. colemani* and *H. satomiae* will have to await the acquisition of collection material. *Hippocampus nalu* also shares with *H. satomiae* double spines above the eye. *Hippocampus colemani* differs in having a low and rounded coronet (versus distinct coronet in *H. satomiae*) and reduced spines at key placements on the trunk and tail ridges (Kuiter 2003), whereas *H. satomiae* differs in extremely small size (14 mm SL), distinct raised coronet with laterally expanded anterior and posterior flanges, and reduced ossification of the inferior and ventral trunk ridges (Lourie and Kuiter 2008) similar to that observed in *H. bargibanti* and *H. denise* (Gomon and Kuiter 2009).

**Figure 7.**
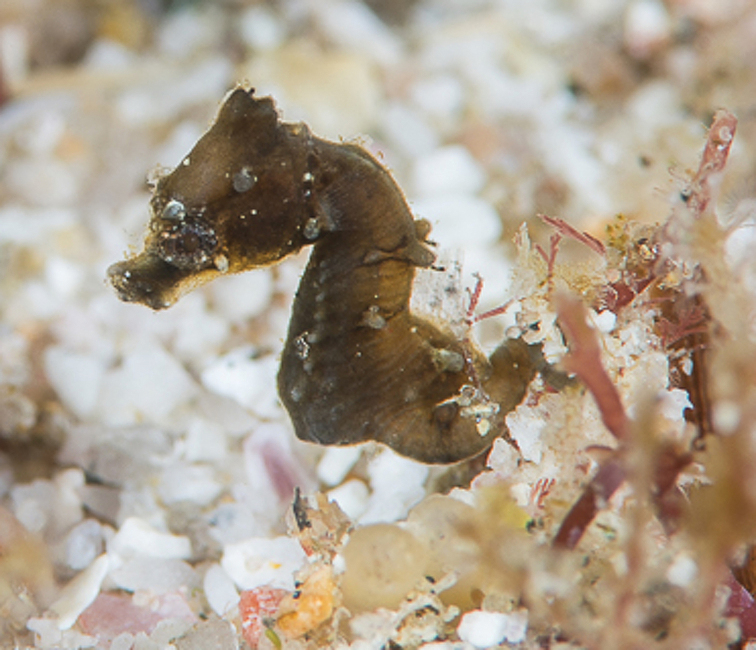
*Hippocampus nalu* in situ, juvenile, approximately 10 mm SL, Sodwana Bay, South Africa at 14 m depth (photograph Richard Smith / oceanrealmimages.com).

**Figure 8.**
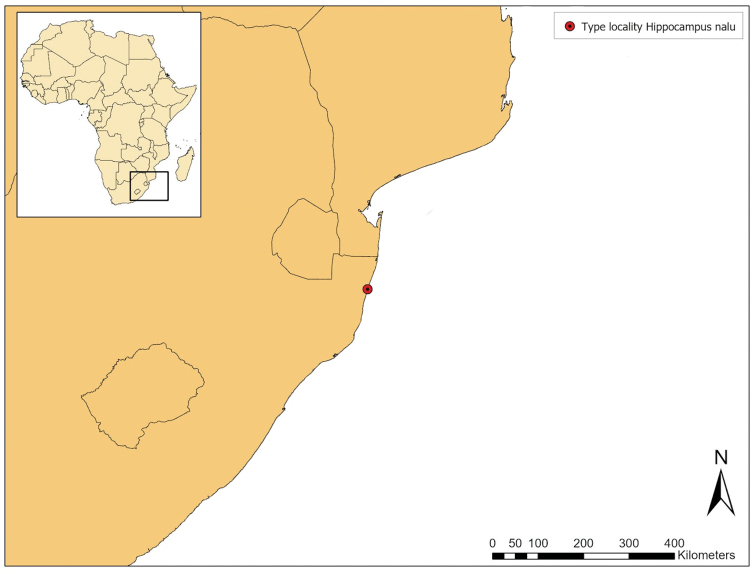
Distribution of *Hippocampus nalu*. Type locality in red.

## Discussion

Here, we consider *Hippocampus nalu* as a valid species due to its genetic and morphological uniqueness; however, a more comprehensive phylogenetic study is necessary to elucidate its evolutionary relationship to its pygmy congeners. Using micro-computed tomography scans, we have identified key diagnostic characters in the highly distinct spine morphology of the two pairs of bilateral wing-like spines present on the first and second superior trunk ridges that differentiate *H. nalu* from the morphologically similar *H. japapigu* and *H. pontohi*. *Hippocampus nalu*, *H. colemani*, *H. japapigu*, *H. pontohi*, *H. satomiae*, and *H. waleananus* are united by numerous morphological synapomorphies and morphologically conserved to the extent that it is difficult to distinguish the species based on external appearance alone, and appear to form a natural grouping in comparison to the distinct *H. bargibanti* and *H. denise*. However, based on examined specimens so far, *Hippocampus nalu*, *H. japapigu*, and *H. pontohi* can be primarily distinguished from one another by features of the distinct morphology and number of wing-like spines on the superior trunk ridges as revealed by μCT scans (Short et al. 2018). *Hippocampus colemani*, *H. satomiae*, and *H. waleananus* appear to have wing-like spines but their morphology in the anterior segments of the trunk have yet to be determined even though the number of spines have been surmised (Short et al. 2018). Secondary characteristics that distinguish between the species include the presence or absence of snout spines, single or double eye spines dorsally, small eye spines posteriorly, sixth and eighth superior trunk ridge spines, and the number of large subdorsal spines.

Seven new species of pygmy seahorses have been officially described and named within the first two decades of the 21^st^ century (Kuiter 2003; Lourie and Randall 2003; Lourie and Kuiter 2008; Gomon and Kuiter 2009; Short et al. 2018). Previously, *H. bargibanti* was the only pygmy seahorse known to science, having been described in 1970 (Whitley 1970). The occurrence of a new species and first record of a pygmy seahorse, *H. nalu*, in South Africa and the Western Indian Ocean is not unexpected but amazing nevertheless since it has been 50 years to date since the discovery of *H. bargibanti*. Fundamental information on the distribution and abundance of pygmy seahorses is still relatively limited relative to the body of literature for the non-pygmy seahorse species. Therefore, we strongly anticipate additional species of pygmy seahorses to be discovered in the Western Indian Ocean by future ichthyofaunal surveys as this region is not as well documented as the Central and Western Indo-Pacific.

## Supplementary Material

XML Treatment for Hippocampus nalu

## Supplementary materials

Supplementary material 1 Genetic distance analysis (uncorrected p distances) of COI sequence data from *H. nalu*, *H. bargibanti*, *H. denise*, *H. japapigu*, and *H. pontohi* Data type Genetic data https://binary.pensoft.net/file/411698This dataset is made available under the Open Database License (http://opendatacommons.org/licenses/odbl/1.0/). The Open Database License (ODbL) is a license agreement intended to allow users to freely share, modify, and use this Dataset while maintaining this same freedom for others, provided that the original source and author(s) are credited.Graham Short, Louw Claassens, Richard Smith, Maarten De Brauwer, Healy Hamilton, Michael Stat, David Harasti
